# Anti-Vaccine Attitudes among Adults in the U.S. during the COVID-19 Pandemic after Vaccine Rollout

**DOI:** 10.3390/vaccines10060933

**Published:** 2022-06-10

**Authors:** Jasmin Choi, Sarah A. Lieff, Gabriella Y. Meltzer, Margaux M. Grivel, Virginia W. Chang, Lawrence H. Yang, Don C. Des Jarlais

**Affiliations:** 1 Department of Social and Behavioral Sciences, School of Global Public Health, New York University, New York, NY 10003, USA; lieffs01@nyu.edu (S.A.L.); gm2477@nyu.edu (G.Y.M.); mg5757@nyu.edu (M.M.G.); vc43@nyu.edu (V.W.C.); ly1067@nyu.edu (L.H.Y.); ddj2@nyu.edu (D.C.D.J.); 2 Department of Population Health, Grossman School of Medicine, New York University, New York, NY 10003, USA; 3 Department of Epidemiology, Mailman School of Public Health, Columbia University, New York, NY 10032, USA; 4 Department of Epidemiology, School of Global Public Health, New York University, New York, NY 10003, USA

**Keywords:** COVID-19, vaccine, anti-vaccine, United States

## Abstract

Even though vaccination is the most effective measure against COVID-19 infections, vaccine rollout efforts have been hampered by growing anti-vaccine attitudes. Based on current knowledge, we identified three domains (beliefs, discrimination, and news) as our correlates of primary interest to examine the association with anti-vaccine attitudes. This is one of the first studies to examine key correlates of anti-vaccine attitudes during the critical early stages of vaccine implementation in the United States. An online survey was administered in May 2021 to a non-representative, nationally based sample of adults (N = 789). Using multivariable logistic regression analysis, we found that individuals who expressed worry about COVID-19 (OR = 0.34, 95% CI 0.21, 0.55) and had greater knowledge of COVID-19 (OR = 0.50, 95% CI 0.25, 0.99) were less likely to hold anti-vaccine attitudes. Conversely, individuals who held stigmatizing views of COVID-19 (OR = 2.47, 95% CI 1.53, 3.99), had experienced racial discrimination (OR = 2.14, 95% CI 1.25, 3.67) and discrimination related to COVID-19 (OR = 2.84, 95% CI 1.54, 5.24), and who had been watching Fox News (OR = 3.95, 95% CI 2.61, 5.97) were more likely to hold anti-vaccine attitudes. These findings suggest COVID-19 beliefs, experiences of discrimination, and news sources should be considered when designing targeted approaches to address the anti-vaccine movement.

## 1. Introduction

Since the emergence of the novel SARS-CoV-2 coronavirus in December 2019, COVID-19 has infected more than 129 million individuals worldwide, including 2.9 million deaths by the beginning of April 2021 [1,2]. In the U.S. alone, there were over 549,000 deaths during the same period [2,3]. As a result, most states in the U.S. issued statewide stay-at-home orders and mask mandates to curb the rapid spread of COVID-19 infection and its devastating effects. In December 2020, the COVID-19 vaccine rollout in the U.S. began for the most high-risk and vulnerable groups. All adults in the U.S. have been eligible for the vaccine since April 2021. While personal protective measures, such as face masks, frequent hand washing, and social distancing can reduce the risk of infection, vaccination has been shown to be the most effective tool against the spread of COVID-19. Still, vaccine hesitancy and anti-vaccine attitudes remain relatively common in the public discourse. Despite the wide availability of vaccines by April 2021, vaccination rates quickly stagnated, with between 45% and 56% of adults across states receiving at least one dose as of May 2021—the same period in which our study was conducted [4]. With low rates of vaccine uptake being partially attributable to the anti-vaccine movement, it is crucial that we address the public’s attitudes and intentions toward COVID-19 vaccination.

In light of the anti-vaccine movement becoming a growing public health concern, general vaccine hesitancy and anti-vaccination attitudes have been extensively studied. These concepts are distinct in that those who are vaccine-hesitant may accept, delay, or refuse certain vaccines due to fear, lack of confidence, mistrust, or other sociocultural beliefs or norms [5,6,7,8], while anti-vaccination attitudes are largely fueled by misinformation or a lack of knowledge, leading to the refusal of vaccinations based on the belief that vaccinations cause adverse effects [5,8,9,10]. In the last decade, the anti-vaccination movement has been thriving due to a surge of vaccine misinformation being shared online [8,11]. Particularly during the COVID-19 pandemic, addressing and combatting these anti-vaccine attitudes should be prioritized. Globally, anti-vaccine attitudes and vaccine hesitancy impacted COVID-19 vaccine uptake [12,13,14]. In the U.S., the anti-vaccine movement continues to be a major challenge amid public health efforts to control the COVID-19 pandemic.

The growing trend of anti-vaccine attitudes and vaccine hesitancy in the context of COVID-19 has been well documented [15,16,17], with most studies prior to the vaccine rollout reporting that social and behavioral factors, such as knowledge, risk perception, beliefs, and stigma are highly correlated with vaccine intention [18,19,20,21,22,23,24]. The Health Belief Model is a prominent theoretical framework that explains the behavioral intentions and motivators for individuals engaging in health-promoting behavior to avoid diseases [25]. Similarly, attitudes toward COVID-19 vaccines can be understood using the Health Belief Model, where an individual’s perceived susceptibility and severity, as well as benefit, barriers, self-efficacy, and cues to action can lead to the intent to receive vaccines to avoid COVID-19 disease [26].

In contrast, health illiteracy and inadequate knowledge on COVID-19 have been shown to be associated with negative views toward vaccination [27]. As demonstrated with other highly stigmatized illnesses, such as substance use disorders, HIV, or severe mental health disorders, stigmatizing beliefs and attitudes toward specific health conditions may delay or discourage help-seeking behaviors [28,29,30,31]. In particular, one study suggested that worrying about contracting COVID-19 is strongly associated with perceived severity of, and stigmatizing attitudes towards, COVID-19, which may impact vaccine uptake [32]. Moreover, studies on COVID-19 vaccine hesitancy conducted before widespread availability of the COVID-19 vaccine have found that political ideology is associated with vaccination attitudes such that people are less willing to be vaccinated if they have conservative political leanings or rely on conservative leaning news sources [18,19,20,21,22,33,34,35]. Yet, little is known about whether COVID-19 related stigma, knowledge, and bias remain strongly associated with anti-vaccine attitudes after the start of vaccine rollout in the U.S.

Findings pertaining to the sociodemographic correlates of vaccine attitudes have been inconsistent across the literature. Most notably, studies of racial differences in vaccine attitudes have been inconclusive, with some studies reporting no association between race and vaccine hesitancy [21,22], as well as others reporting that individuals who identified as Black were less willing to receive COVID-19 vaccines in the future [18,19,22,23,24,33]. Research supports that, given the historical and ongoing landscape of racism in the U.S., there exists a level of mistrust and low confidence in the healthcare system among Black and other racial minority communities [36,37]. With multiple studies providing evidence to support that racial discrimination may contribute to medical mistrust, it is plausible that these experiences lead to a decrease in preventative health behaviors, such as vaccination [38,39,40,41,42]. Namely, one study observed that experiencing racial discrimination in general has predicted COVID-19 vaccine hesitancy [43]. Recent evidence shows that a greater rate of discriminatory experiences related to COVID-19 were observed across all racial and ethnic minority groups compared to Whites [44]. In one national poll, Black and Asian Americans were more likely to experience racial/ethnic discrimination since the COVID-19 outbreak than their white and Hispanic counterparts [45]. To better understand anti-vaccine attitudes, we consider experiences of racial/ethnic discrimination as one of our correlates of primary interest, rather than focusing solely on race/ethnicity. Inconsistent findings pertaining to the association between COVID-19 vaccine hesitancy and other sociodemographic factors, including gender, education levels, and employment status, have also been reported and merit further investigation [18,19,20,21,22,23,24,33,34,35].

While previous studies have summarized the public perception of COVID-19 vaccines and vaccination intentions prior to vaccine rollout in the U.S., only a handful of studies [35,43,46] in which post-vaccine rollout was conducted have examined vaccine hesitancy and willingness to be vaccinated. These studies support that conservative political leanings [35] and experiences of racial discrimination [43] are associated with a higher level of vaccine hesitancy, and that individuals who identify as Asian have the highest vaccine acceptance rate [46]. Importantly, these studies were conducted during the initial phase of vaccine rollout between December 2020 and February 2021 when vaccines were only available for high-risk groups. Our study aims to fill this gap by assessing anti-vaccine attitudes among U.S. adults after the COVID-19 vaccine rollout. This study includes measures of stigma, discrimination, and preferred news sources to understand the sociopolitical context that may influence vaccine intentions during the pandemic.

Based on research conducted prior to the wide-scale vaccine rollout, our study identified COVID-19 beliefs, experiences of discrimination, and news sources as primary correlates of anti-vaccine attitudes to examine whether the associations remain strong even after the vaccine rollout was well underway. Our study uses a nationally based sample from an online survey to examine public attitudes toward vaccination during the COVID-19 pandemic. To our knowledge, this is the first study to characterize and identify correlates of anti-vaccine attitudes during the post-vaccine rollout period when all adults in the U.S. were eligible for their first and second doses of COVID-19 vaccines. Since the initial launch of the vaccination program, there have been many studies demonstrating the safety, efficacy, and effectiveness of available COVID-19 vaccines [47,48,49]. Prior to our study, the CDC released new guidance that fully vaccinated individuals no longer needed to wear masks or practice social distancing outdoors or in most indoor settings, excluding places required by federal, state, and local regulations [50,51]. These COVID-19 related developments make our survey data collection time critically relevant, particularly for this study that assesses anti-vaccine attitudes in real time during the post-vaccine rollout. We conducted this study to provide more context for anti-vaccine attitudes by identifying significant correlates of anti-vaccine attitudes during the COVID-19 vaccine rollout in the U.S. Since the anti-vaccine movement is considered to be a consequence of sociodemographic and behavioral factors, it is crucial to examine how anti-vaccine attitudes manifest among individuals.

This study further contributes to the literature by proposing an innovative 16-item scale designed for anti-vaccine attitudes multidimensionally by capturing COVID-19 attitudes, beliefs, and knowledge. In previous studies, vaccine hesitancy and anti-vaccine attitudes were measured with either a single-question or six-item scale of a broad scope [20]. Instead, our scale includes specific items that are narrow in scope to capture the sociopolitical context of the COVID-19 pandemic accurately, such as attitudes toward American and Chinese governments, worries about new variants, and mask mandates, among others. With this novel scale, our study examines whether anti-vaccine attitudes are associated with sociodemographic characteristics, COVID-19-related beliefs, health status, interpersonal contact with COVID-19 and Chinese individuals, discrimination experiences, and news sources. Given plateaued vaccination rates, such information will be helpful when developing and implementing strategies to combat the anti-vaccine movement.

## 2. Materials and Methods

### 2.1. Study Design

This study uses the second wave of survey data from a research study examining knowledge and attitudes toward HIV/AIDS, SARS, and COVID-19. The second survey wave was administered from May 21 to 26 May 2021 using Amazon’s Mechanical Turk (MTurk) platform. This crowdsourcing platform allows researchers to conduct rapid online surveys with a diverse global workforce [52]. The eligibility criteria for the study included U.S.-based respondents who were 18 years or older and had at least a 90% approval rate for a minimum of 500 previously completed MTurk tasks. A detailed description of the study’s survey design, data collection, and data quality control have been described in detail in previous studies [32,53]. Due to the mechanisms of the MTurk platform, self-selection is unavoidable as MTurk participants self-select into the platform and then self-select into a study [54]. To reduce selection bias, we did not provide specific details of the study, such as mentioning anti-vaccine attitudes. Instead, we provided a broad description of the study purpose, which was to learn more about public knowledge and attitudes towards HIV/AIDS, SARS, and COVID-19. The target sample size for the survey was 1200. A convenience sampling method was used for data collection. This study utilizes a survey dataset from a non-representative nationally based sample (N = 1001). A final analytic sample of 789 respondents was selected for this analysis after excluding 212 respondents due to missing and unknown data. All survey respondents provided online informed consent and were compensated for approximately ten minutes of their time to complete the survey. All study procedures were approved by the New York University Institutional Review Board (IRB-FY2020-4402, approved 5 August 2020).

### 2.2. Measures

The main outcome was vaccine attitudes, which was dichotomized as pro-vaccine and anti-vaccine attitudes using the median split of the composite scale score. The COVID-19 attitudes, beliefs, and knowledge scale was constructed from 16 items that were each scored from 1 to 4 (strongly agree, agree, disagree, and strongly disagree), with reverse coding for specific items as shown in Table A1. Across our sample, composite scale scores ranged from 16 to 64 and were dichotomized at the median of 35. Lower scores reflected positive attitudes toward vaccination or agreement with evidence-based public health approaches to the COVID-19 pandemic (“pro-vaccine”). Scores above the median were indicated as “anti-vaccine.” The reliability of the scale was more than acceptable (Cronbach’s α = 0.81).

For correlates of primary interest, we measured COVID-19 beliefs (stigma, worry, and knowledge), experiences of discrimination, and news sources. Survey items assessing COVID-19 stigma, worry, and knowledge were adapted from previous studies on the stigmatization of HIV/AIDS and SARS and their associated high-risk groups [55]. A five-item COVID-19 stigma composite was used to measure whether respondents had COVID-19 related stigmatizing attitudes. This composite was adapted from another study that examined the stigmatization of AIDS and SARS [55] and has been described in detail in a previous study [53]. This stigma composite had high reliability as measured by Cronbach’s α of 0.80 and 0.72 for the AIDS and SARS stigma surveys, respectively. A past study conducted a principal components analysis for the COVID-19 stigma scale, which revealed a 1-factor solution with excellent reliability (Cronbach’s α = 0.90) [53]. To measure COVID-19 worry, respondents were asked how worried they were about contracting COVID-19, and the responses were dichotomized (“Very worried”/“Somewhat worried” or “Not at all worried”). To measure COVID-19 knowledge, the respondents were asked how much they have heard about COVID-19, and responses were then dichotomized (“A great deal” or “Some”/“Not much”).

Respondents were asked about their experiences of stigmatization or discrimination because of their race/ethnicity and related to COVID-19. Items included the following: “Have you experienced stigmatization or discrimination because of your race/ethnicity?” and “Have you experienced stigmatization or discrimination related to COVID-19?” The responses were dichotomized to capture any experiences of stigmatization or discrimination compared to having no experiences (“Yes, a lot”/“Yes, some” or “No”).

Respondents were asked to identify their preferred news sources based on a provided list. These responses were categorized as “Social Media” (Facebook, Twitter), “Publicly-funded News” (PBS, NPR), and “Commercial TV News” (CNN, MSNBC, ABC, CBS, NBC), along with the New York Times and Fox News remaining as separate news sources. Fox News was reported separately from commercial TV news because its media bias and its effect on consumers’ COVID-19 attitudes have been studied previously [56,57,58,59]. While Fox News appeals to more conservative leaning consumers, the New York Times appeals to more liberal leaning consumers [60]. Moreover, the New York Times won the 2021 Pulitzer Prize for Public Service for its coverage of the COVID-19 pandemic [61]. Previous studies have examined the differences between Fox News and the New York Times in different contexts [60,62,63].

Other potential correlates, including health status, interpersonal contact with COVID-19 and Chinese individuals, and sociodemographic characteristics were also measured. Health status was assessed by asking participants to self-identify the existence of “underlying conditions, such as diabetes, overweight, heart disease, lung/breathing diseases, which could make COVID-19 diseases more severe.” Probable depression was assessed using the Patient Health Questionnaire-9 (PHQ-9) at a cut-off score of five or greater, and probable anxiety was measured using the Generalized Anxiety Disorder-2 (GAD-2) at a cut-off score of 3 [64]. Interpersonal contact was assessed by asking respondents whether they knew someone who “had COVID-19” and “became seriously ill or died from COVID-19” and whether they knew someone who “identified as a Chinese individual.” The three interpersonal contact items were dichotomized into “No Contact” and “Contact” based on the range of responses from “definitely yes” to “definitely not.” Self-reported demographic characteristics included gender, age group, race/ethnicity, education level, and employment status.

### 2.3. Statistical Analyses

A univariate analysis was conducted to describe the characteristics of the final study sample (N = 789) of the study. Crude odds ratios were calculated using univariable logistic regression models to assess the main effects of variables on anti-vaccine attitudes, followed by multivariable logistic regression to calculate adjusted odds ratios.

We tested for multi-collinearity of the variables using a correlation matrix, variance inflation factor, and Akaike information criteria. Anxiety and depression scores, and scores on the two COVID-19 contact items (i.e., contact with someone who had COVID-19, or severe COVID-19) were highly correlated and collinear, respectively. As such, anxiety and contact with those who had been infected with COVID-19 were both dropped as variables from the analysis. The final model was then used in a multivariable logistic regression to calculate the adjusted odds ratios for anti-vaccine attitudes. We also evaluated the predictive ability of the primary correlates for estimating anti-vaccine attitudes by conducting separate logistic regression analyses for each (COVID-19 beliefs, discrimination, and news), as shown in Table A2. We calculated the McFadden pseudo R^2^ to measure the model fit, and we also conducted the likelihood ratio test to test the model’s predictive ability [65]. Additionally, we calculated the area under the curve (AUC) of the receiver operating characteristic (ROC) to indicate the effect size for multivariable logistic regression [66]. Statistical significance was determined based on *p* < 0.05 for all analyses. All statistical analyses were performed in STATA 17 [67].

## 3. Results

Table 1 shows descriptive statistics of the final sample (N = 789). Most respondents were male (59.8%), 35 years or older (56.5%), non-Hispanic White (66.9%), had at least a college degree (53.4%), and were employed full-time (77.8%).

The crude and adjusted odds ratios of anti-vaccine attitudes are shown in Table 2. All correlates of primary interest—COVID-19 beliefs (stigma, worry, and knowledge), experiences of discrimination, and news sources—were significantly associated with our main outcome of having anti-vaccine attitudes. We found that the three correlates of primary interest (beliefs, discrimination, and news) significantly improved the predictive power of our model in estimating anti-vaccine attitudes (likelihood ratio test, *p* < 0.001). In the crude model, the AUC-ROC was 0.686. In the adjusted model, the AUC-ROC was 0.869, which indicated that about 87% of anti-vaccine attitudes would be correctly predicted by the model.

In the adjusted model, those who held stigmatizing views of COVID-19 were significantly more likely to have anti-vaccine attitudes (OR = 2.47, 95% CI 1.53, 3.99). In contrast, the odds of having anti-vaccine attitudes were significantly lower for those worried about COVID-19 (OR = 0.34, 95% CI 0.21, 0.55) and those with knowledge of COVID-19 (OR = 0.50, 95% CI 0.25, 0.99) after adjusting for other covariates.

In the adjusted model, the odds of having anti-vaccine attitudes were significantly higher for those who had experienced stigmatization or discrimination based on race or ethnicity (OR = 2.14, 95% CI 1.25, 3.67) and related to COVID-19 (OR = 2.84, 95% CI 1.54, 5.24).

In the crude analysis, the odds of having anti-vaccine attitudes were significantly higher for individuals who identified social media, such as Facebook or Twitter, as their preferred news source, but were no longer significant in the adjusted analysis. While social media as a news source was not significantly associated with anti-vaccine attitudes, other news sources had significant associations in the adjusted model. In the adjusted model, the odds of having anti-vaccine attitudes were significantly lower for those who received news from publicly funded news sources, such as PBS or NPR (OR = 0.59, 95% CI 0.37, 0.95), commercial TV news, such as CNN, MSNBC, ABC, CBS, or NBC (OR = 0.40, 95% CI 0.26, 0.60), and the New York Times (OR = 0.57, 95% CI 0.37, 0.86). In the adjusted model, those who consumed Fox News were significantly more likely to have anti-vaccine attitudes (OR = 3.95, 95% CI 2.61, 5.97).

We found mixed results for other potential correlates, such as sociodemographic and health characteristics, including interpersonal contacts. In an unadjusted model, individuals who identified as non-Hispanic Black (OR = 1.80, 95% CI 1.05, 3.08) and Hispanic (OR = 2.99, 95% CI 2.01, 4.43) were significantly more likely to have anti-vaccine attitudes compared to non-Hispanic White individuals. However, these associations were no longer significant after adjusting for all the main exposures. Instead, Asian individuals were significantly less likely to have anti-vaccine attitudes (OR = 0.45, 95% CI 0.21, 0.96) compared to non-Hispanic Whites in the adjusted model. There were no significant associations between anti-vaccine attitudes and gender, age group, education level, or employment status in the adjusted model.

In the adjusted model, those with probable depression were significantly more likely to have anti-vaccine attitudes (OR = 1.90, 95% CI 1.24, 2.92). Those with underlying high-risk medical conditions for severe COVID-19 illness were significantly more likely to have anti-vaccine attitudes compared to those without medical conditions in the unadjusted model, but after adjusting for other factors, this association was no longer significant. In the adjusted model, those who had contact with individuals who had been seriously ill or had died from COVID-19 were significantly less likely to have anti-vaccine attitudes (OR = 0.59, 95% CI 0.37, 0.93). Individuals who had interpersonal contact with Chinese individuals were significantly less likely to have anti-vaccine attitudes in the crude analysis, but no significant association was observed in the adjusted analysis.

## 4. Discussion

As of April 2022, approximately 66% of the U.S. population are fully vaccinated, and approximately 18 states are reporting at a low rate below 60% [2,68]. Even a year after the wide-scale vaccine rollout, the vaccine rate still remains low despite a growing need for booster shots. Given the stagnated vaccination rates, we must address and mitigate the anti-vaccine movement by examining significant correlates of anti-vaccine attitudes. Our study fills the gap in the literature by exploring public attitudes toward vaccination in the post-vaccine rollout period during the COVID-19 pandemic. COVID-19 infections were already widespread globally at the time of data collection, and the vaccine rollout was well underway for several months in the U.S. Since this is one of the first studies to examine anti-vaccine attitudes after all U.S. adults became eligible for the COVID-19 vaccine, our findings provide more context to lagging vaccination rates despite widespread availability and accessibility. This study examines the associations of anti-vaccine attitudes with three correlates of primary interest (COVID-19 beliefs, experiences of discrimination, and news sources), along with other correlates of interests, in a non-representative, nationally-based sample from an online survey in May 2021. Our study shows that the correlates of primary interest remain strongly associated with anti-vaccine attitudes even after COVID-19 vaccination was readily available to the general U.S. public.

From global literature, it is evident that greater knowledge of COVID-19 is associated with a greater likelihood of adopting preventative measures [27,69,70,71]. Our findings are consistent with the current literature that suggests that health literacy and risk perception contribute to health behavior adoption [27,69,70,71]. We found that those who were worried and reported being more knowledgeable about COVID-19 were less likely to have anti-vaccine attitudes. These findings suggest that being informed about the risks of COVID-19 and being afraid or concerned about COVID-19 may contribute to a willingness to be vaccinated. Similarly, those who had contact with someone with severe COVID-19 were less likely to have anti-vaccine attitudes, suggesting that close contact with illness may have affected risk perception. Our findings align with the current evidence that risk perceptions play a significant role in vaccine uptake [19,26,33,72,73]. These results can be explained by the Health Belief Model [25], which demonstrates that one’s perception of risk and severity of illness can lead to recommended health-related actions. When an individual perceives the susceptibility and severity of COVID-19 risks, these beliefs can lead to taking a preventative measure or adopting a health-promoting behavior, such as being willing to receive a COVID-19 vaccine. As explained by the Health Belief Model, we can infer that the individuals who reported being more knowledgeable about COVID-19 may also experience greater perceived susceptibility. Likewise, being worried about COVID-19 and being in contact with someone with severe COVID-19 illness may lead to greater perceived susceptibility. In turn, these increased levels of perceived susceptibility can activate the perceived threat of COVID-19. We can see that these factors contribute to having pro-vaccine attitudes (or being less likely to hold anti-vaccine attitudes). These attitudes toward COVID-19 vaccines can be explained as antecedents to health-promoting action. To increase COVID-19 vaccine uptake and address anti-vaccine attitudes, we can leverage perceptions of risk for COVID-19 as part of a broader public health and vaccination campaign. We can frame messages to emphasize the personal risks of COVID-19 disease and any other health risks that come from not being vaccinated. At the same time, public health messaging needs to be carefully tailored so as to not cause panic, fear, or anxiety among the general public and cause unintended harmful consequences of increased depression or increased stigmatization.

In addition to risk perception, social and political factors should also be considered when addressing anti-vaccine attitudes. Our findings confirmed that those who endorsed COVID-19 stigma and watched Fox News were more likely to hold anti-vaccine attitudes. While the exact relationship between news consumption, COVID-19 stigma, and vaccine attitudes is unknown, two potential and inter-related pathways to anti-vaccine attitudes may be emerging: the “news–stigma–vaccine pathway” and the “news–government distrust–vaccine pathway”. First, research suggests that the consumption of conservative-leaning news is strongly associated stigmatizing attitudes towards COVID-19 [53], and our findings show, in turn, that COVID-19 stigma is predictive of anti-vaccination attitudes. Second, recently published research demonstrates that consumption of conservative-leaning news is strongly associated with the belief that the CDC exaggerated the danger of COVID-19 in the U.S. [74], and previous studies lend support to the influence of perceived government trustworthiness on vaccine decision-making [75,76]. While hypothesizing on the direction of causation in these emerging potential pathways to anti-vaccination attitudes is beyond the scope of the current study, these proposed relationships merit further investigation.

Similar to the mixed results seen in the current body of literature, we also detected no significant associations between most racial/ethnic groups and anti-vaccine attitudes in our adjusted model. That being said, our results showing that Asian individuals are less likely to have anti-vaccine attitudes align with findings from previous studies that show that Asian groups had higher COVID-19 vaccine acceptance and less vaccine hesitancy compared to other racial/ethnic groups [33,46,77,78]. According to recent data, the overall rate of receiving at least one dose of COVID-19 vaccination for Asian Americans was 84%, which was higher than for Hispanic (64%), White (62%), and Black Americans (57%) [79]. Although we did not detect significant differences across racial/ethnic groups for anti-vaccine attitudes, with the exception of identifying as Asian, we suspect that experiences of racial/ethnic discrimination are indicative of racial disparities in vaccine uptake.

Rather than focusing solely on race/ethnicity, we identified having experiences with discrimination based on race/ethnicity and related to COVID-19 to be correlates of primary interest. Our finding is in agreement with another study that found that experiences of racial discrimination in general—not related to COVID-19—had predicted vaccine hesitancy [43]. Furthermore, the experiences of discrimination related to COVID-19 can address how social context behind COVID-19 stigma is evolving. Experiencing discrimination can lead to internalized stigma, which can impact health-seeking behaviors, adherence to care, and preventative measures, including vaccination [28,29,30,31,42,80,81]. Previous studies noted that COVID-19 related stigma and discrimination are likely caused by multiple socio-ecological and structural factors, and experiences with COVID-19 discrimination are ever-changing among people of Asian descent, European travelers, immigrants, healthcare workers, and those with long-haul COVID-19 [82,83]. Our findings emphasize that sociodemographic factors, such as race/ethnicity, are not the sole factor that predict vaccine attitudes. Instead, we focus on individuals’ experiences, beliefs, and attitudes related to COVID-19 as the correlates of primary interest for having anti-vaccine attitudes during the vaccine rollout. Similar to recent COVID-19 literature, we also found gender, education levels, and employment status to be not strongly associated with COVID-19 anti-vaccine attitudes [18,19,20,21,22,23,24,33,34,35].

Our study has several limitations. First, our sample is not nationally representative; our study findings may therefore not be generalizable. Generally, MTurk participants tend to be younger and more educated than the general population [54]. Likewise, our sample had fewer older adults and was more highly educated. Past studies show that older adults and more highly educated individuals tend to be less vaccine hesitant [13,84]. For our analysis, we controlled for sociodemographic factors in the multivariable analyses to account for this limitation. Second, since our study is based on cross-sectional data, causal relationships between anti-vaccine attitudes and correlating factors cannot be established. We would note that there is a high likelihood of bidirectional causation among some of the variables. For example, while consuming certain media sources might lead to endorsing specific attitudes, having the specific attitudes might lead one to consume certain media. We would need data from multiple time points to examine these possibilities in detail. For this report, we conducted analyses beyond simple bivariable analyses, but emphasized that our multivariable analyses are exploratory. Third, there may be self-report bias from our study survey. Due to the highly politicized nature of COVID-19 and the anti-vaccine movement, we suspect that social desirability effects may have biased respondents’ reports of anti-vaccine attitudes, stigma, experiences with discrimination, and even certain preferred news sources. However, it should be noted that we observed that almost half of our respondents reported having stigmatizing views of COVID-19. Lastly, there may have been other unobserved covariates that might have introduced additional residual confounding. Future studies should include individual determinants such as religious or political affiliation, household income, and zip code to identify structural determinants at city and state levels such as vaccine accessibility, availability, incentives, and other vaccine rollout measures to increase uptake. Another consideration is to examine the recent trend of anti-vaccine attitudes toward COVID-19 booster shots. All adults became eligible for booster shots in November 2021, yet state efforts to enforce boosters vary widely. Building on our findings, future studies should also compare public attitudes toward vaccines in general in the post-COVID-19 era.

## 5. Conclusions

The results from our study have profound policy implications related to implementing effective vaccination outreach strategies geared toward population characteristics, attitudes, and behaviors associated with anti-vaccine attitudes. Our findings add to the current evidence that considering the role of negative and positive emotions and experiences can help foster vaccine confidence and address anti-vaccine attitudes [85]. Addressing negative emotions and experiences tied to COVID-19 is needed for certain communities and groups since they have experienced discrimination and stigma during the pandemic [45,82,85]. A more targeted community-based campaign to address negative experiences of stigmatization and discrimination can help earn community members’ trust and confidence in the uptake of COVID-19 vaccination.

Moreover, tackling misinformation and sensationalized headlines on social media and news media can help combat the anti-vaccine movement [86]. Increasing COVID-19 literacy and knowledge contributes to health-promoting behavior. Our findings provide compelling evidence for a tailored approach to effective vaccination campaigns through targeted efforts toward individuals who prefer certain news sources, such as Fox News, and those who experienced stigmatization or discrimination based on race/ethnicity and related to COVID-19. Therefore, COVID-19 vaccination efforts must focus on increasing trust among groups who may have experienced discrimination in healthcare and those who consume conservative leaning news outlets. With variants emerging globally, it is imperative that we improve existing vaccination campaigns to expedite COVID-19 vaccine uptake, including the booster dose.

## Figures and Tables

**Table 1:** Descriptive statistics of the non-representative national sample, N = 789.

**Sociodemographic**	**N (%)**
Gender	
Female	317 (40.18)
Male	472 (59.82)
Age	
18–24 years	25 (3.17)
25–34 years	318 (40.30)
35–44 years	248 (31.43)
45–54 years	109 (13.81)
55+	89 (11.28)
Race	
Non-Hispanic White	528 (66.92)
Non-Hispanic Black	61 (7.73)
Hispanic	146 (18.50)
Asian	54 (6.84)
Education	
High School or Below	91 (11.53)
Some College	157 (19.90)
College Degree (BA/BS)	421 (53.36)
Graduate School	120 (15.21)
Employment	
No employment	86 (10.90)
Part-time	89 (11.28)
Full-time	614 (77.82)
**COVID-19 beliefs**	**N (%)**
Holds stigmatizing view of COVID-19	
No	428 (54.25)
Yes	361 (45.75)
Worried about COVID-19	
Not at all worried	190 (24.08)
Somewhat/very worried	599 (75.92)
Knowledge of COVID-19	
Not much/Some	103 (13.05)
A great deal	686 (86.95)
**Health**	**N (%)**
Underlying conditions increasing risk for severe COVID-19 illness	
No	492 (62.36)
Yes	297 (37.64)
Depression	
No probable depression	373 (47.28)
Probable depression	416 (52.72)
Anxiety	
No anxiety	502 (63.62)
Probable anxiety	287 (36.38)
**Interpersonal Contact**	**N (%)**
Contact with COVID-19	
No contact	172 (21.80)
Contact	617 (78.20)
Contact with Severe COVID-19	
No contact	450 (57.03)
Contact	339 (42.97)
Contact with Chinese individuals	
No contact	159 (20.15)
Contact	630 (79.85)
**Discrimination**	**N (%)**
Have you experienced stigmatization or discrimination because of your race/ethnicity?	
No	503 (63.75)
Yes, a lot/some	286 (36.25)
Have you experienced stigmatization or discrimination related to COVID-19?	
No	559 (70.85)
Yes, a lot/some	230 (29.15)
**News Sources**	**N (%)**
Social Media News (Facebook, Twitter)	
No	271 (34.35)
Yes	518 (65.65)
Public Funded News (PBS, NPR)	
No	608 (77.06)
Yes	181 (22.94)
Commercial TV News (CNN, MSNBC, ABC, CBS, NBC)	
No	249 (31.56)
Yes	540 (68.44)
New York Times	
No	491 (62.23)
Yes	298 (37.77)
Fox News	
No	511 (64.77)
Yes	278 (35.23)

**Table 2:** The regression models predicting anti-vaccine^†^ attitudes, Non-Representative National sample, N = 789.

Predictors	Crude OR (95% CI)	Adjusted OR (95% CI)
**Sociodemographic**		
Gender		
Female	Ref.	Ref.
Male	1.29 (0.97, 1.71)	1.14 (0.76, 1.70)
Age		
18–24 years	Ref.	Ref.
25–34 years	1.31 (0.57, 2.99)	0.84 (0.27, 2.65)
35–44 years	2.04 (0.88. 4.73)	1.78 (0.55, 5.76)
45–54 years	1.37 (0.57, 3.31)	1.26 (0.37, 4.33)
55+	1.22 (0.50, 3.02)	1.86 (0.53, 6.55)
Race		
Non-Hispanic White	Ref.	Ref.
Non-Hispanic Black	1.80 (1.05, 3.08) *	1.41 (0.69, 2.87)
Hispanic	2.99 (2.01, 4.43) ***	0.74 (0.40, 1.35)
Asian	0.68 (0.38, 1.21)	0.45 (0.21, 0.96) *
Education		
High School or Below	Ref.	Ref.
Some College	0.77 (0.46, 1.31)	1.33 (0.69, 2.55)
College Degree (BA/BS)	1.75 (1.10, 2.76) *	1.38 (0.76, 2.50)
Graduate School	1.65 (0.95, 1.86)	1.38 (0.67, 2.87)
Employment		
No employment	Ref.	Ref.
Part-time	1.82 (0.97, 3.40)	1.59 (0.71, 3.53)
Full-time	2.84 (1.73, 4.64) ***	1.71 (0.90, 3.26)
**COVID-19 beliefs**		
Holds stigmatizing view of COVID-19		
No	Ref.	Ref.
Yes	6.00 (4.40, 8.18) ***	2.47 (1.53, 3.99) ***
Worried about COVID-19		
Not at all worried	Ref.	Ref.
Somewhat/very worried	1.08 (0.78, 1.49)	0.34 (0.21, 0.55) ***
Knowledge of COVID-19		
Not much/Some	Ref.	Ref.
A great deal	0.19 (0.11, 0.31) ***	0.50 (0.25, 0.99) *
**Health**		
Underlying conditions increasing risk for severe COVID-19 illness		
No	Ref.	Ref.
Yes	2.26 (1.68, 3.03) ***	0.95 (0.60, 1.50)
Depression		
No probable depression	Ref.	Ref.
Probable depression	3.43 (2.56, 4.60)***	1.90 (1.24, 2.92)**
Anxiety		
No anxiety	Ref.	
Probable anxiety	3.87 (2.84, 5.28) ***	
**Interpersonal Contact**		
Contact with COVID-19		
No contact	Ref.	Ref.
Contact	0.46 (0.33, 0.66) ***	0.59 (0.37, 0.93) *
Contact with Severe COVID-19		
No contact	Ref.	
Contact	1.40 (1.05, 1.85) *	
Contact with Chinese individuals		
No contact	Ref.	Ref.
Contact	0.40 (0.28, 0.58) ***	0.95 (0.57, 1.59)
**Discrimination**		
Experienced stigmatization or discrimination because of your race/ethnicity		
No	Ref.	Ref.
Yes, a lot/some	4.96 (3.60, 6.83) ***	2.14 (1.25, 3.67) **
Experienced stigmatization or discrimination related to COVID-19		
No	Ref.	Ref.
Yes, a lot/some	8.66 (5.89, 12.72) ***	2.84 (1.54, 5.24) **
**News Sources**		
Social Media News (Facebook, Twitter)		
No	Ref.	Ref.
Yes	2.23 (1.65, 3.01) ***	1.19 (0.78, 1.80)
Public Funded News (PBS, NPR)		
No	Ref.	Ref.
Yes	0.31 (0.21, 0.44) ***	0.59 (0.37, 0.95) *
Commercial TV News (CNN, MSNBC, ABC, CBS, NBC)		
No	Ref.	Ref.
Yes	0.49 (0.36, 0.67) ***	0.40 (0.26, 0.60) ***
New York Times		
No	Ref.	Ref.
Yes	0.60 (0.45, 0.80) **	0.57 (0.37, 0.86) **
Fox News		
No	Ref.	Ref.
Yes	5.86 (4.21, 8.15) ***	3.95 (2.61, 5.97) ***

*p < 0.05, **p < 0.01, ***p < 0.001. ^†^ Vaccine attitudes scale is dichotomized at the median into two groups: pro-vaccine and anti-vaccine. Table 2 shows outcome of anti-vaccine attitudes.

## Data Availability

The data presented in this study are available upon request from the corresponding author.

## Appendix A

**Table A1:** Anti-Vaccine Attitudes Scale.

**Item—Positively Scored**
It is important to get a lot of people vaccinated so that we can go back to normal life. Overall, the U.S. government has handled the COVID-19 pandemic well, for its citizens. Overall, the Chinese government has handled the COVID-19 pandemic well, for its citizens. The government should it easier to get vaccinated by providing easy appointments, transportation, and paid time off. People who don’t get vaccinated risk getting infected and then infecting others. I am worried about the new variants to the COVID-19 virus. Getting enough people vaccinated so that mask requirements could be reduced was a major accomplishment for the United States.
**Item—Reverse Scored (r)**
8. I believe that the dangers of COVID-19 have been greatly exaggerated. (r) 9. I do not like vaccines in general. (r) 10. I do not trust pharmaceutical companies. (r) 11. People like me have been mistreated by medical authorities. (r) 12. Even if I got infected, I do not think I would get seriously ill from COVID-19. (r) 13. No one in my family has or is likely to get seriously ill from COVID-19. (r) 14. The economic impact of the lockdowns in the US has been worse than the impact of COVID-19 disease. (r) 15. Mask mandates have been a violation of personal rights. (r) 16. The vaccines were developed too quickly to know if they are safe. (r)

## Appendix B

**Table A2:** The regression models of primary correlates predicting anti-vaccine^†^ attitudes, Non-Representative National sample, N = 789.

Predictors	Model 1 Adjusted OR (95% CI)	Model 2 Adjusted OR (95% CI)	Model 3 Adjusted OR (95% CI)	Model 1 + 2+3 Adjusted OR (95% CI)
**COVID-19 beliefs**				
Holds stigmatizing view of COVID-19				
No	Ref.			Ref.
Yes	6.88 (4.81, 9.83) ***			2.72 (1.77, 4.17) ***
Worried about COVID-19				
Not at all worried	Ref.			Ref.
Somewhat/very worried	0.42 (0.29, 0.63) ***			0.41 (0.26, 0.63) ***
Knowledge of COVID-19				
Not much/Some	Ref.			Ref.
A great deal	0.33 (0.19, 0.57) ***			0.50 (0.26, 0.94) *
**Discrimination**				
Experienced stigmatization or discrimination because of your race/ethnicity				
No		Ref.		Ref.
Yes, a lot/some		1.99 (1.33, 2.97) **		1.97 (1.25, 3.11) **
Experienced stigmatization or discrimination related to COVID-19				
No		Ref.		Ref.
Yes, a lot/some		5.53 (3.49, 8.76) ***		3.18 (1.85, 5.48) ***
**News Sources**				
Social Media News (Facebook, Twitter)				
No			Ref.	Ref.
Yes			1.77 (1.25, 2.51) **	1.16 (0.78, 1.72)
Public Funded News (PBS, NPR)				
No			Ref.	Ref.
Yes			0.43 (0.229, 0.65) ***	0.58 (0.36, 0.91) *
Commercial TV News (CNN, MSNBC, ABC, CBS, NBC)				
No			Ref.	Ref.
Yes			0.40 (0.28, 0.57) ***	0.42 (0.28, 0.61) ***
New York Times				
No			Ref.	Ref.
Yes			0.67 (0.48, 0.95) *	0.62 (0.42, 0.91) *
Fox News				
No			Ref.	Ref.
Yes			6.02 (4.22, 8.59) ***	4.29 (2.89, 6.38) ***

* *p* < 0.05, ** *p* < 0.01, *** *p* < 0.001. ^†^ Vaccine attitudes scale is dichotomized at the median into two groups: pro-vaccine and anti-vaccine. Appendix Table A1 shows outcome of anti-vaccine attitudes. Note: McFadden’s pseudo R^2^ for Model 1 = 0.163; McFadden’s pseudo R^2^ for Model 2 = 0.150; McFadden’s pseudo R^2^ for Model 3 = 0.183; McFadden’s pseudo R^2^ for Model 1 + 2 + 3 = 0.310.
